# Genetics of Dothistromin Biosynthesis of *Dothistroma septosporum*: An Update

**DOI:** 10.3390/toxins2112680

**Published:** 2010-11-05

**Authors:** Arne Schwelm, Rosie E. Bradshaw

**Affiliations:** 1 Bio-Protection Research Centre, Institute of Molecular BioSciences, Massey University, Palmerston North, New Zealand; 2 Department of Plant Biology & Forest Genetics, Swedish University of Agricultural Sciences, Box 7080, S-750 07 Uppsala, Sweden; Email: arne.schwelm@vbsg.slu.se

**Keywords:** Dothideomycete, mycotoxin, gene cluster, pine needle blight, *Mycosphaerella pini*, aflatoxin, sterigmatocystin

## Abstract

Dothistroma needle blight is one of the most devastating fungal pine diseases worldwide. The disease is characterized by accumulation in pine needles of a red toxin, dothistromin, that is chemically related to aflatoxin (AF) and sterigmatocystin (ST). This review updates current knowledge of the genetics of dothistromin biosynthesis by the *Dothistroma septosporum* pathogen and highlights differences in gene organization and regulation that have been discovered between the dothistromin and AF/ST systems. Some previously reported genes are promoted or demoted as ‘dothistromin genes’ based on recent research. A new dothistromin gene, *norB,* is reported, and evidence of dothistromin gene homologs in other Dothideomycete fungi is presented. A hypothesis for the biological role of dothistromin is outlined. Finally, the impact that the availability of the *D. septosporum* genome sequence will have on dothistromin research is discussed.

## 1. Introduction

*Dothistroma septosporum* (teleomorph *Mycosphaerella pini*) and *D. pini* (no known teleomorph) are ascomycete pathogens in the class Dothideomycetes that cause Dothistroma needle blight (DNB) [1]. DNB is an economically important foliar disease affecting coniferous trees, particularly pines, that can cause needle death, premature defoliation and, in severe cases, tree mortality [2]. While *D. septosporum* has a world-wide distribution and a broad host range [1,6], *D. pini* has so far only been reported from the north-central U.S. and several parts of Europe [1,3,4,5]. DNB has been prevalent since the 1960s in pine plantation forests, particularly in the Southern hemisphere where both the pathogen and pine host are exotics. But during the last two decades the incidence of DNB has increased dramatically in the northern hemisphere including areas with native hosts [3,6,7,8]. These epidemic outbreaks are associated with wetter summers that may be a consequence of climate change [8,9]. Both *D. septosporum* and *D. pini* are listed as quarantine organisms and are therefore subject to special monitoring in many countries [10]. The potential geographic range determined to be suitable for DNB extends beyond the current known range of this disease, and predictions that DNB might become more prevalent in the future are already being borne out by recent first disease reports in the Baltics [2,7]. 

The most characteristic symptom of DNB is bright red banding in infected needles, earning the disease the alternative name ‘red band’ disease. The red bands are due to accumulation of a polyketide derived toxin, dothistromin, produced by the *Dothistroma* pathogens [11]. Dothistromin is produced and secreted by the fungus in culture as well as *in planta*. Some isolates produce more toxin in culture than others [12] but it is not known if these are also higher-producers *in planta*. Dothistromin has been isolated from diseased needles and was suspected to play a role in the disease process [13]. But the red toxin bands are not always present in infected needles and it was shown that a dothistromin-deficient *D. septosporum* strain was able to infect radiata pine [14]. Dothistromin does have a broad toxicity to many types of organisms [15] and it has been proposed to confer a competitive advantage for *D. septosporum* against other microorganisms rather than being a pathogenicity factor for DNB [14]. 

One of the interesting aspects of the dothistromin toxin is its close structural similarity to versicolorin B. Versicolorin is a biosynthetic precursor of the potent natural carcinogens aflatoxin (AF) and sterigmatocystin (ST). Previous studies identified eleven *D. septosporum* genes with high identities to AF/ST biosynthesis genes (Table 1). These are believed to be dothistromin biosynthesis genes; this has been confirmed experimentally for three of them [16,17,18]. The dothistromin biosynthesis genes are located in at least three genomic fragments, interspersed with apparently functionally unrelated genes [18]. This is in contrast to AF/ST genes that are organized in a continuous gene cluster [19,20].

This review describes the current knowledge of the genetics of dothistromin biosynthesis in *D. septosporum* and brings together several new discoveries and hypotheses. In view of recent research, we question whether some genes previously designated as dothistromin genes should be demoted. Conversely we suggest promoting some previously ‘non-dothistromin’ genes to dothistromin gene status. An additional dothistromin biosynthetic gene, *norB*, is reported that may represent a fourth genomic fragment of dothistromin biosynthesis genes. Some orthologues of dothistromin/AF/ST genes have been newly identified in other Dothideomycete species, suggesting a capacity for biosynthesis of related secondary metabolites. A hypothesis is outlined about a biological function of dothistromin in competition with other microorganisms. Many questions remain about the genetics and regulation of dothistromin biosynthesis and the role of dothistromin: We discuss to what extent the genome sequence of *D. septosporum* will enable these questions to be answered.

**Table 1:** *D. septosporum* genes and their predicted gene products, with amino acid identities to *A. nidulans* (ST) and *A. parasiticus* (AF) gene products. Horizontal lines separate known mini-clusters. *D. septosporum* genes experimentally verified as dothistromin genes are in bold.

Gene	Predicted Size (aa)	Putative Gene Product Function	ST Gene Ortholog	Identity aa (%)	AF Gene Ortholog	Identity aa (%)
***dotA***	263	Versicolorin reductase	*stcU*	79.1	*aflM*	80.2
*dotB*	414	Oxidase	*stcC*	24.0		
*dotC*	602	MFS transporter			*aflT*	31.2
*dotD*	322	Thioesterase	*stcA*	37.9	*aflC*	34.8
***pksA***	2399	Polyketide synthase	*stcA*	57.0	*aflC*	54.8
*cypA*	511	Averufin monooxygenase	*stcB*	59.8	*aflV*	59.3
*avfA*	301	Oxidase	*stcO*	43.7	*aflI*	47.8
*epoA*	420	Epoxide hydrolase				
*moxA*	626	Monooxygenase	*stcW*	59.0	*aflW*	55.1
DS31	231	Translation elongation factor	*stcT*	41.1		
*hexA*	(321)	Fatty acid synthase (partial)	*stcJ*	41.3	*aflA*	48.8
*hypC*	262	Anthrone oxidase	*stcM*	47.9	*hypC*	35.2
***vbsA***	643	Versicolorin B synthase	*stcN*	69.1	*aflK*	72.0
*norB*	392	Norsolorinic acid reductase	*stcV*	43.4	*aflF*	60.7

## 2. The Dothistromin Toxin

The DNB pathogens *D. septosporum* and *D. pini* are both producers of dothistromin. Dothistromin is also produced by some other fungal pathogens in the class Dothideomycetes. These include *Passalora arachidicola* (previously called *Cercospora arachidicola*) [21], various *Cercospora* species such as *C. ferruginea*, *C. fusca*, *C. microsora*, *C. rosicola*, *C. rubi* and *C. smilacis* [22,23] and the larch pathogen *Mycosphaerella laricina* [24]. 

**Figure 1:**
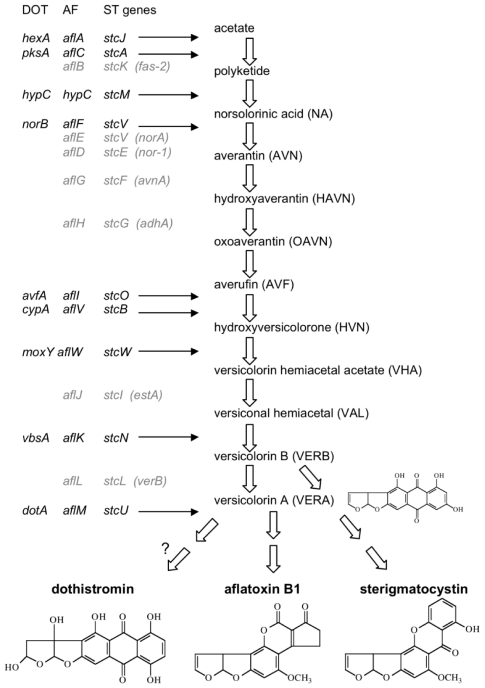
Simplified biosynthetic pathway for aflatoxin (AF) and sterigmatocystin (ST) and predicted pathway for dothistromin (DOT) showing key intermediates and chemical structures of pathway products. Probable sites of action of the currently known dothistromin genes (left), and their AF and ST orthologs, are indicated. Some other biosynthetic genes expected to be present in *D. septosporum* are indicated in grey, with old AF gene names in parenthesis for clarification.

The ‘dothistromin’ extracted from *D. septosporum* is actually a mixture of dothistromin, which accounts for 80–90% of the mixture, and deoxydothistromin [25]. These compounds have a difuranoanthraquinone structure, as shown by mass spectrometry and NMR [11]. It was noted that dothistromin belongs to the same group of metabolites as the aflatoxins, sterigmatocystin and versicolorins as they share a common furobenzofuran moiety [25], although dothistromin is unusual in having a 1,4-hydroxylation arrangement in the anthraquinone ring system (Figure 1). AF is the best studied fungal polyketide-derived metabolite and is produced by many different species of *Aspergillus* [26]. Only recently has the synthesis of AF been confirmed to be produced outside the genus *Aspergillus*, by *Fusarium kyushuense* [27]. The best known ST producer is *A. nidulans*, but ST is produced by a large number of phylogenetically diverse fungi in addition to many *Aspergillus/Emericella* species [26].

Dothistromin has a broad range of toxicity against bacterial, fungal, plant and animal cells. The mode of action of the dothistromin toxin is not clarified and appears to have different effects on different cell types. The toxicity and possible mode of action of dothistromin was covered in an earlier review [6] and is thought to involve reductive oxygen activation. In pines the host defense contributes to the symptoms seen in Dothistroma needle blight. Injection of purified dothistromin into pine needles mimicks the disease symptoms, but is also accompanied by a strong host defense response. Accumulation of benzoic acid, made by the plant, occurs in high enough concentrations to be inhibitory to *D. septosporum*, but at the same time is toxic to plant cells. Purified dothistromin is quickly broken down in the needle tissue, with only 10–20% of the toxin remaining after 24 h. As the necrotic lesion continues to expand after most of the dothistromin has been destroyed it was suggested that most of the needle damage is due to the plant's defense [13,28].

## 3. The Genetic Basis of Dothistromin Biosynthesis

### 3.1. AF/ST-like Genes and Gene Organization

The genes for a secondary metabolite pathway in filamentous fungi are generally clustered at a single genomic locus and located in telomere-proximal regions of fungal genomes [29,30]. The synthesis of AF requires around 25 genes, clustered together in a 70 kb region of the genome [31]. Characterization of the ST cluster in *A. nidulans* showed that most of the ST genes are orthologs of AF genes, but that they are arranged in a different order within the cluster [19,32]. 

Analysis of AF/ST gene clusters from a range of *Aspergillus* species suggested that the ST-type cluster is ancestral to the AF-type. Cary and Ehrlich [33] proposed an ancestral basal cluster of genes (*pksA*, *hexA*, *hexB*, and *nor-1*) required to form and stabilize the initial polyketide, along with some regulatory genes (*aflR*, *aflJ*). These authors speculated that duplication and recruitment of genes encoding polyketide-modifying enzymes subsequently occurred, followed by purifying selection and some gene loss. Indeed there is considerable evidence for duplication of genes in metabolic gene clusters [33,34] and for the occurrence of transposable elements and/or repeated sequences that suggest a mechanism whereby movement of sets of genes may have occurred during the evolution of gene clusters [35,36]. A more detailed study of sequence variation in ST/AF clusters identified seven modules of duplicated genes, and recombination blocks, supporting the hypothesis that gene duplication, vertical transmission and rearrangement have been important in the evolution of these gene clusters [37,38].

**Figure 2:**
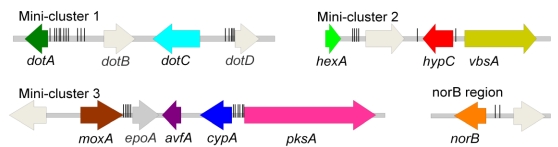
Arrangement of dothistromin genes (colored) and other genes (grey) in 3 mini‑clusters and a *norB* region. Each of the regions shown is flanked by other ‘non‑dothistromin’ genes [18] apart from regions adjacent to *hexA* and *norB* that are currently unknown. Arrows indicate the direction of transcription and vertical bars indicate positions of the sequence TCGN_5_CGR, a binding site for AflR in *Aspergillus* spp.

The identification of dothistromin genes was facilitated by extensive knowledge of AF biosynthesis genes in *Aspergillus* spp. The similarity of the chemical structure of dothistromin to the AF/ST biosynthesis precursor versicolorin, the detection of AF/ST biosynthetic intermediates in dothistromin‑producing *D. septosporum* cultures, and ^13^C-NMR analysis of dothistromin [39] suggested a similar biosynthetic pathway for AF and dothistromin. Using degenerate PCR primers and hybridization probes based on genes required for AF biosynthesis, dothistromin biosynthetic genes were identified [17,18]. Somewhat surprisingly, despite their strong similarities to AF/ST genes, dothistromin biosynthesis genes in *D. septosporum* are separated into several mini-clusters (Figure 2) and not organized in a continuous gene cluster as seen for AF/ST, although all genes appear to be located on a 1.3-Mb chromosome [18]. Analysis of dothistromin biosynthesis genes in the dothistromin producing fungal peanut pathogen *Passalora arachidicola* revealed a very similar organization for the dothistromin genes in at least three mini-clusters [40].

Besides the biosynthetic genes for dothistromin there are other known examples of fungal secondary metabolite biosynthesis pathways with genes located at spatially separated genomic regions. These include T-toxin biosynthesis in *Cochliobolus heterostrophus* [41], trichothecene biosynthesis in *Fusarium* spp [42] and aflatrem biosynthesis genes in *A. flavus* [43]. In the aflatoxin biosynthesis gene cluster from *A. ochraceoroseus*, a species more related to *A. nidulans* than to *A. flavus*, the gene organization is similar to the *A. nidulans* ST cluster, except for the position and orientation of a six‑gene block; however additional genes such as *aflP* and *aflQ* that are required for biosynthesis of AF from ST are speculated to be located elsewhere in the genome [44].

Homologs of AF/ST biosynthesis genes can be found in many species. At least some of the genes required for production of aflatoxins are present in species of *Aspergillus* not known to be able to make aflatoxins or its precursors, such as *A. terreus* or *A. fumigatus* [29,45]. Homologs of AF biosynthetic genes are involved in biosynthesis of a red pigment in *Nectria haematococca* [46]. Some *Lactobacillus brevis* strains have a homolog of the AF *adhA* dehydrogenase, which was able to convert AF precursor 5-hydroxyaverantin to averufin in feeding studies. The same reaction is found in aflatoxin biosynthesis, although *L. brevis* does not produce AF or any precursors [47]. 

Besides AF and ST, *Aspergillus* species are able to produce other polyketide-derived secondary metabolites and some of these metabolites, such as monodictyphenone in *A. nidulans*, involve homologs of ST/AF/dothistromin genes. Production of monodictyphenone and related emodin derivatives was discovered in a strain with deletion of the *cclA* gene that is involved in epigenetic regulation [48]. Although the monodictyphenone genes are clustered, they are also dispersed with genes not involved in monodictyphenone biosynthesis. Furthermore the identified cluster contains homologs of the *pksA*, *dotA* and DS31 dothistromin genes (*mdpG, mdpC* and *mdpJ* respectively).

A search of the currently available genomes of other Dothideomycete species with the *D. septosporum* dothistromin genes revealed many homologs (Table 2). These homologs were dispersed on different scaffolds with no evidence of clustering. Some genes such as polyketide synthases are known to be present in many copies in fungal genomes and to have diverse functions [49]; hence it is not surprising to find genes similar to dothistromin *pksA*. Other genes involved in AF/ST biosynthesis also occur as replicated families: *A. flavus* and *A. nidulans* have 13 and 10 copies of the monooxygenase gene *aflW/stcW* respectively [38] and, as expected, the Dothideomycetes also have genes matching the *D. septosporum* monooxygenase ortholog *moxA*. Some AF/ST genes are usually only present in 1–3 copies in the *Aspergillus* genomes [38]; the Dothideomycetes shown in Table 2 have matches to *D. septosporum* orthologs of some of these ‘low copy number’ genes (*dotA* and *cypA*) but not to others (*avfA*).

Further searching of three Dothideomycete genomes with ‘low-copy number’ *A. parasiticus* genes *aflP (omtA), aflX (ordB)* (required for ‘late’ stages of AF biosynthesis) and *aflR* (regulatory gene) showed potential orthologs for some of these genes in each species (Table 2). However products of the *aflP* (predicted methyltransferase) and *aflX* (predicted oxidoreductase) homologs could have general functions in these fungi. LC-MS analysis of metabolites produced by *Mycosphaerella graminicola* in culture did not reveal any dothistromin production by this species. But it is possible that some of the dothistromin/AF-like genes function in biosynthesis of polyketide‑derived secondary metabolites in these or other Dothideomycete species.

**Table 2:** Dothistromin/AF-like genes in some Dothideomycete genomes. Predicted amino acid sequences of *D. septosporum* (Ds) dothistromin genes and selected *A. parasiticus* (Ap) aflatoxin genes were compared to genome sequences of the three fungi shown (held at the Joint Genome Institute; http://www.jgi.doe.gov). E-values and amino acid identities (% aa ID) are based on blastp searches against filtered gene models (proteins). Only the top hit is shown for each. Where e < 10^−20^ the protein ID and corresponding gene location (chromosome or scaffold number) are shown.

Gene Product	*M. graminicola*		*M. fijiensis*		*C. heterostrophus*	
	e-Value(% aa ID)	Protein ID (chromosome)	e-Value(% aa ID)	Protein ID (scaffold)	e-Value(% aa ID)	Protein ID (scaffold)
DsDotA	0 (71)	87994 (11)	0 (72)	145765 (10)	0 (65)	25675 (10)
DsDotC	0 (57)	100062 (4)	0 (57)	58758 (8)	0 (53)	82192 (3)
DsPksA	0 (33)	96592 (11)	0 (34)	216850 (10)	0 (34)	30478 (10)
DsCypA	1e-45 (27)	32226 (1)	0 (34)	190367 (8)	0 (38)	63323 (5)
DsAvfA	[2e-09]		no hits		[3e-19]	
DsMoxA	0 (71)	60715 (7)	0 (69)	56721 (7)	0 (66)	13307 (9)
DS31	0 (46)	55916 (2)	0 (48)	70922 (2)	0 (54)	117471 (8)
DsHexA	0 (40)	108403 (3)	0 (40)	212667 (12)	0 (40)	102681 (5)
DsHypC	[9e-08]		9e-23 (35)	34418 (8)	[1e-13]	
DsVbsA	0 (42)	71382 (4)	0 (44)	86877 (7)	0 (42)	113329 (25)
DsNorB	0 (59)	105021 (7)	0 (61)	36962 (9)	0 (58)	109470 (15)
ApAflP	7e-37 (25)	25671 (2)	2e-25 (30)	99112 (8)	2e-30 (26)	62952 (5)
ApAflX	0 (47)	96127 (10)	no hits		0 (36)	95438 (27)
ApAflR	[2e-07]		7e-31 (11)	198930 (8)	[5e-16]	

### 3.2. Dothistromin Genes

#### 3.2.1. Functionally Analysed Dothistromin Genes

The *pksA, vbsA* and *dotA* genes of *D. septosporum* have been experimentally confirmed to be involved in the biosynthesis of dothistromin [16,17,18]. All three genes were initially identified on genomic clones clustered alongside other putative dothistromin genes [18]. The *pksA* gene encodes a polyketide synthase (PKS) with 55% amino acid identity to *A. parasiticus* AflC [17]. The predicted PksA protein contains the same set of functional domains found in AF/ST PKS proteins of *Aspergillus* spp., except it has three tandem ACP domains, in contrast to the single ACP in *A. parasiticus* AflC and two ACPs in *A. nidulans* StcA. A *pksA* gene replacement in *D. septosporum* resulted in a mutant which did not produce detectable amounts of dothistromin [17]. However when supplied with norsolorinic acid or versicolorin A, this strain was able to produce dothistromin, supporting NMR results that suggested a similar synthesis of AF/ST and dothistromin [39].

The *vbsA* gene is predicted to encode versicolorin B synthase on the basis of similarity to AflK (72% amino acid identity) and StcN (69% identity). Replacement of the *vbsA* gene in *D. septosporum* led to loss of dothistromin production, confirming its role in dothistromin biosynthesis. When complemented with a *vbsA* wild type gene the phenotype was rescued [18].

The *dotA* gene encodes a ketoreductase with 80% amino acid identity to AflM (Ver-1) in *A. parasiticus* and was also shown, by gene replacement, to be necessary for dothistromin biosynthesis [16]. The *aflM* gene encodes a 28-kDa NADPH-dependent reductase that, with the enzymes AflN and AflY [50], converts versicolorin A to demethylsterigmatocystin [51]. A similar function is suggested for DotA. A biosynthetic pathway was proposed for dothistromin that involves epoxidation of versicolorin A and subsequent opening of the epoxide resulting in a secondary alcohol, followed by DotA-catalyzed reduction to yield 5,8-dihydroxyanthraquinone, a compound that has been identified in *D. septosporum* [51,52]. It is suggested that instability of the epoxide and secondary alcohol intermediates led to the accumulation of versicolorin A that occurred in the *dotA* mutant [16].

#### 3.2.2. Other Putative Dothistromin Genes

Additional genes have been identified that are believed to be involved in dothistromin biosynthesis on the basis of their homology to AF/ST biosynthesis genes and, in many cases, by their co-regulation with the characterized *pksA, vbsA* and *dotA* genes [18]. For example, *hexA*, *cypA*, *moxA* and *avfA* are homologs of well characterized AF/ST biosynthesis genes that are all involved in the synthesis of versicolorin precursors in AF/ST biosynthesis (Table 1, Figure 1, Figure 2). We propose that these *D. septosporum* genes catalyse the same biosynthesis steps as their AF/ST homologs and they will not be discussed further here. However, there are six other genes shown in Table 1 (*dotB, dotC, dotD, epoA, DS31, hypC*) that are considered putative dothistromin genes but for which their function, if any, is less well defined. The current status of these genes with regard to their involvement in dothistomin biosynthesis will be discussed here. In addition a recently identified new putative dothistromin gene, *norB*, is described.

The *dotB* gene is predicted to encode a putative oxidase whose function is unknown. In liquid culture *dotB* shows a similar expression pattern to other dothistromin genes [18] although a statistically relevant co-regulation pattern was only observed in comparison to the *avfA* gene [53]. There is a *dotB* homolog in the ST cluster (*stcC*) but not the AF cluster, albeit the predicted amino acid identity between DotB and StcC is only 24% and is the lowest shown in Table 1. An *stcC* mutant of *A. nidulans* still produced sterigmatocystin so the function is unknown, although StcC has some similarities to chloroperoxidase enzymes involved in lignin degradation [32]. It seems likely that DotB might not be involved in dothistromin biosynthesis at all and that *dotB* is just another example of an ‘unrelated’ gene associated with a genuine dothistromin gene (*dotA*), which happens to have a similarity to *stcC*.

The *dotD* gene appears to encode a thioesterase (TE) related to polyketide synthase TE domains but shares only 32.5% predicted amino acid identity to the TE domain of the dothistromin PksA. Indeed it shows greater similarity to conidial pigment PKSs, such as *A. fumigatus* PksP/Alb1 (XP_756095.1; 46% aa identity) and *A. nidulans* Ywa1 (Q03149.2; 44% aa identity). Intriguingly, although DotD would seem to be superfluous for dothistromin biosynthesis given the presence of PksA, the expression of *dotD* gene is co-regulated with *pksA* [18]. Thus, like *dotB*, involvement of *dotD* in dothistromin biosynthesis seems unlikely and it may have an unrelated function. The *dotB* and *dotD* genes are divergently transcribed from the adjacent *dotA* and *dotC* genes respectively, and sequences that match binding sites for the AflR AF/ST regulatory protein are present in the shared upstream regions (Figure 2). This, or the physical association with the *dotA* region, may account for the observed co‑regulation of *dotB* and *dotD* with genuine dothistromin genes.

The *dotC* gene that is located between *dotB* and *dotD* might have some function in dothistromin biosynthesis, but in a rather unexpected indirect regulatory role [54]. DotC is predicted to encode a major facilitator superfamily (MFS) transporter. There is no homologous gene in the ST cluster but DotC has 31% identity to the AF cluster gene product AflT (Table 1). Since dothistromin has broad‑spectrum toxicity it was previously speculated that DotC could have a role in dothistromin secretion from the cell and, therefore, in protecting *D. septosporum* from its own toxin, as seen for MFS proteins in some other plant pathogenic fungi [55]. The expression of *dotC in vitro* differed from other putative dothistromin genes by being constitutive [53], which was concordant with a predicted role in toxin secretion. However, a *dotC* disruption mutant of *D. septosporum* revealed that DotC is not essential for dothistromin secretion [54]; as also seen for the *A. parasiticus afl*T gene which is not required for production or secretion of AF [56]. Instead the disruption and over-expression of *dotC* resulted in altered expression of *dotA*, *vbsA* and *pksA* dothistromin genes, with lower expression and 4-5-fold less dothistromin produced in the KO mutant, and higher gene expression and more than 30-fold increase of dothistromin production in the *dotC* over-expressing strain [54]. These results suggest that DotC is involved in regulation of toxin production, by a mechanism which is not yet known. One possibility is that DotC is involved in the transport of dothistromin, or dothistromin precursors, into intracellular compartments. In *A. parasiticus* it is proposed that late stages of AF synthesis occur in small intracellular vesicles termed ‘aflatoxisomes’ and that associated degradative functions occur in vacuoles [57]. We propose that mid to late stages of dothistromin biosynthesis also occur in vesicles as proposed for AF biosynthesis and that this enables *D. septosporum* to tolerate high intracellular toxin concentrations. This is supported by the location of a DotA-GFP fusion protein in vesicles [54]. A DotC-GFP fusion protein was also associated with vesicles and vacuoles, suggesting that DotC may be involved in intracellular transport. However in vacuoles the DotC-GFP was not confined to the periphery as might be expected for a vacuolar transporter [54] hence the role of DotC remains uncertain.

One of the ‘non-dothistromin’ genes, *hypC* (previously called DS12), located in the *D. septosporum vbsA* mini-cluster, should now be promoted to ‘dothistromin gene’ status. It has been re-named *hypC* on the basis of recent work with the *A. parasiticus* ortholog *hypC* [58]. *A. parasiticus* HypC is an anthrone oxidase that catalyzes oxidation of norsolorinic acid anthrone, a precursor of AF and dothistromin. Mutants of *hypC* still produced AF so it was speculated that non-enzymatic oxidation might be an alternative pathway. Orthologous *hypC* genes are also found in gene clusters of fungi for other anthrone derived metabolites, including ST (*stcM*) and monodictyphenone (*mdpD*) [58]. Therefore we propose that *D. septosporum* HypC is involved in the biosynthesis of dothistromin, taking the role HypC has in the biosynthesis of AF. On the basis of alignments with *A. parasiticus*, *A. flavus* and *A. nidulans* homologs, we suggest that *D. septosporum* DS12/HypC may have previously been incorrectly annotated [18] and that only the C-terminal 142 amino acids of the published 262 amino acid sequence are actually present in the gene product.

The DS31 gene is linked to mini-cluster 3 and is about 15 kb upstream of the *moxA* gene shown in Figure 2. DS31 has homologs in the ST gene cluster (*stcT*), as well as in the monodictyphenone cluster (*mdpJ*), of *A. nidulans* [32,48]. The functions of those genes are not known, but they show similarities to translation elongation factor and glutathione S-transferase genes. Deletion of *stcT* in *A. nidulans* results in reduced production of ST [32], and therefore might be involved in ST synthesis.

Positioned between *moxA* and *avfA* in *D. septosporum* is a gene, *epoA*, which does not have a homolog in the AF/ST gene clusters. The predicted product of *epoA* is an epoxide hydrolase. It is feasible that an epoxide hydrolase could be involved in dothistromin biosynthesis if an epoxide intermediate is involved as previously suggested [51]. Although *epoA* is expressed in culture, one of three conserved amino acid positions in the active site of the predicted protein is different to that expected and work is in progress to determine if it is functional. Furthermore, in the related dothistromin-producing fungus *Passalora arachidicola*, an epoxide hydrolase gene similarly placed between putative dothistromin genes is a pseudogene [40]. Further work will determine whether the *D. septosporum* EpoA is involved in dothistromin biosynthesis.

An ortholog of the AF *aflF* (*norB*) gene was identified in *D. septosporum* by degenerate PCR followed by library screening [59]. The predicted gene product had higher amino acid identity (60.7%) to *A. parasiticus* AflF (NorB) than to AflE (NorA) (46%) and the *D. septosporum* gene was therefore designated *norB*. Adjacent to *D. septosporum norB* on the gDNA library clone were three further genes showing no homologies to AF or ST cluster genes, raising the possibility that *norB* represents a fourth region of dothistromin biosynthetic genes in this species. The function of *D. septosporum* *norB* has not been experimentally determined. However the AF genes *norA* and *norB* are predicted to encode NAD+ or NADP+-dependent alcohol dehydrogenases. Their involvement in the biosynthesis of AF has not been confirmed, but it was suggested that they are involved in the conversion of norsolorinic acid (NA) to averantin (AVN) [20]. Deletion of *norA* did not impair the ability to convert NA to AVN [60] or to produce AF [61]. Likewise disruption of the ST ortholog *stcV* in *A. nidulans* did not influence the production of ST [32]. However, a *norB* mutant accumulated immediate precursors of AFG_1_ and is therefore possibly involved in a late stage of AF synthesis [61]. If NorB does have a role in dothistromin biosynthesis in *D. septosporum,* involvement in conversion of NA to AVN would be most likely, since AVN and NA are probable precursors of dothistromin biosynthesis and no AF production has been shown [16].

### 3.3. Regulation of Dothistromin Biosynthesis

The production of dothistromin by *D. septosporum* is affected by the composition of the medium [16]. More dothistromin was produced in media containing glucose, rather than peptone, as carbon source as is also found for AF production in *A. parasiticus*. No dothistromin was produced when ammonium was used as the nitrogen source as also observed for the synthesis of ST in *A. nidulans* [16,62]. Aerated (shaking) cultures produced more dothistromin than static cultures. More dothistromin was detected from *D. septosporum* isolates grown in the dark compared with those grown in the light, although dothistromin is degraded in the light which would have influenced levels.

When the dothistromin gene regions were first identified, putative binding sites for AF regulatory transcription factors were detected. Sequences for the AF/ST pathway specific regulator AflR (5’-TCGN_5_CGR-3’), as well as a variation of this motif (5’-TCGN_11_CGR-3’) are present upstream of most dothistromin genes [16,17,18] (Figure 2). However, these putative binding motifs are also present upstream of many of the genes designated as ‘non-dothistromin’ [18] and whether an AflR-like protein is involved in the regulation of dothistromin genes is unknown. A possible *aflR* homolog was recently identified in a pre-release of the *D. septoporum* genome sequence and it will be interesting to see if this is a functional ortholog of AF/ST *aflR*.

The expression of AF/ST genes and the regulation of AF/ST production in *Aspergillus* has been the subject of many reviews [30,32,63,64,65]. In addition to the pathway-specific regulator AflR, the VelB/VeA/LaeA complex coordinates fungal development and secondary metabolism in *Aspergillus* spp. [66] and it is evident that chromatin-level regulation is a key factor in regulation of AF/ST production [30]. Although dothistromin genes are not organized in a continuous cluster as seen for the AF/ST biosynthesis genes they appear to be co-regulated [18,67]. A specific regulation of the dothistromin genes appears to exist given that genes adjacent to the dothistromin genes in the identified mini-clusters show a different expression [18].

In *Aspergillus* species there is a strong correlation of mycotoxin gene expression and asexual regulation [68]. The ability to produce spores decreases with the ability to produce consecutive precursors of ST in *A. nidulans* [69]. Disruption of the *pksA* gene in *D. septosporum* also resulted in mutants which had decreased sporulation [17]. However, the sporulation rate in the cultured *D. septosporum* wild type and mutants does vary remarkably so this observation might not be an effect of the *pksA* disruption.

An interesting feature of dothistromin biosynthesis is that production of dothistromin and the expression of dothistromin genes are highest at an early stage of growth in culture [18,67]. Highest expression of the dothistromin genes coincided with the beginning of exponential growth. The expression declined in mid-exponential growth, and this was reflected by toxin production, which was also highest at the onset of exponential growth [67]. This was not expected since secondary metabolites are usually produced at a later growth stage in culture, although recently a similar expression pattern of secondary metabolite genes has been reported from the ascomycete *Metarhizium robertsii*, a fungus which is adapted to live both as a saprophyte in the soil and as a wide-host-range pathogen of arthropods [70]. One possible explanation for the early onset of dothistromin biosynthesis is that the regulation is density-dependent. In *Aspergillus* species a density dependent response, associated with the *laeA* gene, has been reported for the development of conidia or sclerotia, as well as for AF/ST production, with low density correlated with AF/ST production in culture [71,72]. Therefore a similar effect might have caused the high expression of dothistromin genes at an early time point in the time course experiments when the density of *D. septosporum* in the culture was still very low. An additional possible explanation for the early onset is that expression in the fragmented dothistromin cluster is not constrained by chromatin-level regulation to the same extent as AF/ST clusters, therefore has a different pattern of regulation. The availability of the *D. septosporum* genome will enable rapid identification of homologs of key AF/ST pathway and master regulatory genes and help to determine how dothistromin biosynthesis is regulated.

## 4. A Hypothesis for a Biological Function of Dothistromin

Fungi expend a great deal of effort in the production of secondary metabolites, such as mycotoxins, and it stands to reason that these products must confer some benefits to them. However, the biological function of secondary metabolites and mycotoxins is often not apparent and is difficult to determine experimentally. Even after decades of research the biological roles of AF and ST are unclear, although numerous hypotheses have been proposed. A current hypothesis is that AF has a role in counteracting oxidative stress [73]. Since dothistromin itself is a potent inducer of reactive oxygen species [74] a different role has to be postulated for dothistromin. 

**Figure 3:**
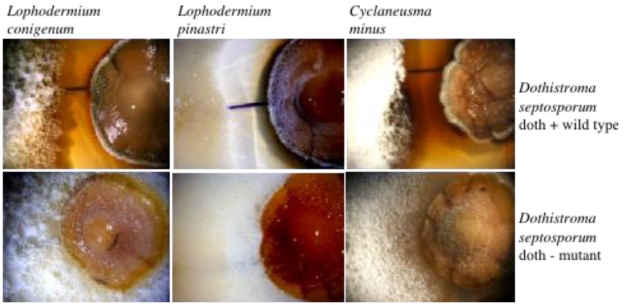
Growth of forest fungi *L. conigenum*, *L. pinastri* and *C. minus* (left colony in each picture) was inhibited in a plate competition assay by dothistromin-producing *D. septosporum* wild type (right hand colony, top row) but not by a dothistromin-deficient strain (NZE7, ∆*pksA*; right hand colony, bottom row). Photographs were taken at the same time points for corresponding doth+ and doth- plates.

We recently proposed an involvement of dothistromin in competition with other microorganisms that inhabit the same ecological niche. This hypothesis is based on the observation that dothistromin‑producing strains of *D. septosporum* are competitive in a plate assay against other fungi. Dothistromin-producing strains inhibited the growth of most fungi tested including the pine dwellers *Lophodermium conigenum, L. pinastri* and *Cyclaneusma minus*, whilst the non-dothistromin producing mutant was overgrown by the competitors [14] (Figure 3). In the natural pine needle environment, there are numerous potential competitors in the form of endophytes and latent pathogens [75,76] and rapid growth of pine needle endophytes can occur when pine tissue is killed [77]. Although it is well documented that dothistromin is produced in pine needles by *D. septosporum*, the timing of this production, and the pattern of growth by the fungus *in planta*, are not known. We previously proposed that *D. septosporum* has a latent phase *in planta* similar to that reported for *Mycosphaerella graminicola* [78] and that concomitant growth and dothistromin production occur after nutrients are released from plant cells killed as part of the disease process, by an as yet unknown mechanism [14]. Further work is required to investigate these important aspects of disease progression *in planta* and to determine whether a competitive advantage of making dothistromin in pine needles can be demonstrated. 

## 5. Outlook for Dothistromin Research with the *D. septosporum* Genome

The availability of the *D. septosporum* genome, being sequenced by the Joint Genome Institute (JGI) as part of the Dothideomycete Comparative Genomics Consortium, will allow the full extent of the dothistromin genes, and how they are arranged, to be determined. As indicated in Figure 1, there are many biosynthetic genes in the AF pathway that we expect to find in the *D. septosporum* genome, such as orthologs of *aflD* (*nor-1*) and *aflG* (*avnA*). Furthermore it is likely that *aflR-* and *aflJ*-like regulatory genes will be found. The genome will also be searched for homologs of late AF pathway genes such as *aflO/P* (*omtA/B*) and *aflQ* (*ordA*) that function after VERA/B in AF biosynthesis. 

The arrangement of these genes—whether they are also in mini-clusters and whether they are on the same 1.3 Mb chromosome—will be of considerable interest and may enable important questions about the evolution of gene clusters to be addressed. The fragmentation seen in *D. septosporum* compared to the clustering of orthologous genes in *Aspergillus* species may help to determine an ‘ancestral’ cluster and to propose mechanisms that led to the gene arrangements seen today. Another question that can be addressed pertains to why the ability to produce specific mycotoxins is so variable between even closely related species. The Dothideomycete class of fungi clearly contain some orthologs of ST/AF/dothistromin genes yet only a handful of them produce these compounds. On a narrower phylogenetic scale, the arrangement of dothistromin genes can be compared with that of the pine pathogen species *Dothistroma pini*. Furthermore it will be possible to determine if there are intraspecific differences between the New Zealand *D. septosporum* strain sequenced by the JGI and current epidemic strains of *D. septosporum* in Canada and Europe. 

With regard to dothistromin biosynthesis, the genome sequence will help to unravel the unusual pattern of regulation seen in *D. septosporum* in culture. Identification of regulatory genes such as homologs of *aflR, veA* and *laeA* will enable genetic dissection of how the expression of dothistromin genes is controlled. Likewise availability of the whole set of putative dothistromin genes, and studies of their co-regulation under different conditions and in different regulatory mutants, will help to define the true extent of the fragmented dothistromin ‘gene cluster’. 

## 6. Conclusions

Our understanding of the genetics of dothistromin biosynthesis is at an important junction. The status of some previously published dothistromin genes has been re-assessed and the ‘gene cluster’ shown to be even more fragmented than previously thought. A new gene, *norB*, was described. The availability of the *D. septosporum* genome sequence will allow identification of the remaining dothistromin genes and analysis of their arrangement in the genome. Many dothistromin-like genes were identified in three other Dothideomycete genomes analyzed. Although in some cases key genes, such as *avfA,* appeared to be missing, it is possible that these species have the potential to produce related metabolites. The biological role of dothistromin in *D. septosporum* is still not known but the current hypothesis is that it enables competition with other microorganisms within the plant tissue.

Keeping in mind that *D. septosporum* is a pathogen causing widespread destruction to forests worldwide, the genetics, regulation and biology of dothistromin biosynthesis are just one aspect of *Dothistroma* research. The *D. septosporum* genome, along with other fungal genomes, will provide opportunities to compare the genetic make-up of pathogens with saprophytes, necrotrophs with biotrophs, and angiosperm pathogens with gymnosperm pathogens. Secondary metabolites, however, may turn out to be important distinguishing features in these comparisons of fungal lifestyles.

## References

[1] Barnes I., Crous P.W., Wingfield B.D., Wingfield M.J. (2004). Multigene phylogenies reveal that red band needle blight of *Pinus* is caused by two distinct species of *Dothistroma, D.septosporum* and *D. pini*. Stud. Mycol..

[2] Watt M.S., Kriticos D.J., Alcaraz S., Brown A.V., Leriche A. (2009). The host spectrum and potential geographic range of Dothistroma needle blight. Forest Ecol. Manag..

[3] Barnes I., Kirisits T., Akulov A., Chhetri D.B., Wingfield B.D., Bulgakov T.S., Wingfield M.J. (2008). New host and country records of the Dothistroma needle blight pathogens from Europe and Asia. Forest Pathol..

[4] Barnes I. (2009). Taxonomy, phylogeny and population biology of the red band needle blight pathogen and related species.

[5] Ioos R., Fabre B., Saurat C., Fourrier C., Frey P., Marçais B. (2010). Development, comparison, and validation of real-time and conventional PCR tools for the detection of the fungal pathogens causing brown spot and red band needle blights of pines. Phytopathology.

[6] Bradshaw R.E. (2004). Dothistroma (red-band) needle blight of pines and the dothistromin toxin: A review. Forest Pathol..

[7] Drenkhan R., Hanso M. (2009). Recent invasion of foliage fungi of pines (*Pinus* spp.) to the Northern Baltics. Forest. Stud..

[8] Welsh C., Lewis K., Woods A. (2009). The outbreak history of Dothistroma needle blight: An emerging forest disease in northwestern British Columbia, Canada. Can. J. Forest Res..

[9] Woods A.J., Coates D.K., Hamann A. (2005). Is an unprecedented Dothistroma needle blight epidemic related to climate change?. BioScience.

[10] EPPO Mycosphaerella dearnessii and Mycosphaerella pini. EPPO Bull..

[11] Bassett C., Buchanan M., Gallagher R.T., Hodges R.L. (1970). A toxic difuroanthraquinone from *Dothistroma pini*. Chem. Ind..

[12] Bradshaw R.E., Ganley R.J., Jones W.T., Dyer P.S. (2000). High levels of dothistromin toxin produced by the forest pathogen *Dothistroma pini*. Mycol. Res..

[13] Shain L., Franich R.A. (1981). Induction of *Dothistroma* blight symptoms with dothistromin. Physiol. Plant Pathol..

[14] Schwelm A., Barron N.J., Baker J., Dick M., Long P.G., Zhang S., Bradshaw R.E. (2009). Dothistromin toxin is not required for dothistroma needle blight in *Pinus radiata*. Plant Pathol..

[15] Stoessl A., Abramowski Z., Lester H.H., Rock G.L., Towers G.H.N. (1990). Further toxic properties of the fungal metabolite dothistromin. Mycopathologia.

[16] Bradshaw R.E., Bhatnagar D., Ganley R.J., Gillman C.J., Monahan B.J., Seconi J.M. (2002). *Dothistroma pini*, a forest pathogen, contains homologs of aflatoxin biosynthetic pathway genes. Appl. Environ. Microbiol..

[17] Bradshaw R.E., Jin H., Morgan B., Schwelm A., Teddy O., Young C., Zhang S. (2006). A polyketide synthase gene required for biosynthesis of the aflatoxin-like toxin, dothistromin. Mycopathologia.

[18] Zhang S., Schwelm A., Jin H., Collins L.J., Bradshaw R.E. (2007). A fragmented aflatoxin-like gene cluster in the forest pathogen *Dothistroma septosporum*. Fungal Genet. Biol..

[19] Brown D.W., Yu J.-H., Kelkar H.S., Fernandes M., Nesbitt T.C., Keller N.P., Adams T.H., Leonard T.J. (1996). Twenty-five coregulated transcripts define a sterigmatocystin gene cluster in *Aspergillus nidulans*. Proc. Natl. Acad. Sci. USA.

[20] Yu J., Bhatnagar D., Cleveland T.E. (2004). Completed sequence of aflatoxin pathway gene cluster in *Aspergillus parasiticus*. FEBS Lett..

[21] Stoessl A. (1984). Dothistromin as a metabolite of *Cercospora arachidicola*. Mycopathologia..

[22] Assante G., Camarda L., Merlini L., Nasini G. (1977). Dothistromin and 2-Epidothistromin from *Cercospora smilacis*. Phytochemistry.

[23] Assante G., Locci R., Camarada L., Merlini L., Nasini G. (1977). Screening of the genus *Cercospora* for secondary metabolites. Phytochemistry.

[24] Assante G. (1985). Isolation and characterization of new dothstromins from *Mycosphaerella laricina* hart. Phytopathol. Med..

[25] Gallagher R.T., Hodges R. (1972). The chemistry of dothistromin, a difuroanthraquinone from *Dothistroma pini*. Aust. J. Chem..

[26] Varga J., Frisvad J.C., Samson R.A. (2009). A reappraisal of fungi producing aflatoxins. World Mycotoxin J..

[27] Schmidt-Heydt M., Häckel S., Rüfer C.E., Geisen R. (2009). A strain of *Fusarium kyushuense* is able to produce aflatoxin B_1_ and G_1_. Mycotoxin Res..

[28] Franich R.A., Carson M.J., Carson S.D. (1986). Synthesis and accumulation of benzoic acid in *Pinus radiata* needles in response to tissue injury with dothistromin, and correlation with resistance of *P. radiata* families to *Dothistroma pini*. Physiol. Mol. Plant Pathol..

[29] Galagan J.E., Calvo S.E., Cuomo C., Ma L.J., Wortman J.R., Batzoglou S., Lee S.I., Basturkmen M., Spevak C.C., Clutterbuck J., Kapitonov V., Jurka J., Scazzocchio C., Farman M., Butler J., Purcell S., Harris S., Braus G.H., Draht O., Busch S., D'Enfert C., Bouchier C., Goldman G.H., Bell-Pedersen D., Griffiths-Jones S., Doonan J.H., Yu J., Vienken K., Pain A., Freitag M., Selker E.U., Archer D.B., Penalva M.A., Oakley B.R., Momany M., Tanaka T., Kumagai T., Asai K., Machida M., Nierman W.C., Denning D.W., Caddick M., Hynes M., Paoletti M., Fischer R., Miller B., Dyer P., Sachs M.S., Osmani S.A., Birren B.W. (2005). Sequencing of *Aspergillus nidulans* and comparative analysis with *A. fumigatus* and *A. oryzae*. Nature.

[30] Georgianna D.R., Payne G.A. (2009). Genetic regulation of aflatoxin biosynthesis: From gene to genome. Fungal Genet. Biol..

[31] Yu J., Chang P.-K., Ehrlich K.C., Cary J.W., Bhatnagar D., Cleveland T.E., Payne G.A., Linz J.E., Woloshuk C.P., Bennett J.W. (2004). Clustered pathway genes in aflatoxin biosynthesis. Appl. Environ. Microbiol..

[32] McDonald T., Noordermeer D., Zhang Y.-Q., Hammond T.M., Keller N.P., Abbas H.K. (2005). The ST cluster revisited: Lessons from a genetic model. Aflatoxin and Food Safety.

[33] Cary J.W., Ehrlich K.C. (2006). Aflatoxigenicity in *Aspergillus*: Molecular genetics, phylogenetic relationships and evolutionary implications. Mycopathologia.

[34] Chang P.-K., Yu J. (2002). Characterization of a partial duplication of the aflatoxin gene cluster in *Aspergillus parasiticus* ATCC 56775. Appl. Microbiol. Biotech..

[35] Tanaka A., Tapper B.A., Popay A., Parker E.J., Scott B. (2005). A symbiosis expressed non-ribosomal peptide synthetase from a mutualistic fungal endophyte of perennial ryegrass confers protection to the symbiotum from insect herbivory. Molec. Microbiol..

[36] Young C.A., Felitti S., Shields K., Spangenberg G., Johnson R.D., Bryan G.T., Saikia S., Scott B. (2006). A complex gene cluster for indole-diterpene biosynthesis in the grass endophyte *Neotyphodium lolii*. Fungal Genet. Biol..

[37] Carbone I., Jakobek J.L., Ramirez-Prado J.H., Horn B.W. (2007). Recombination, balancing selection and adaptive evolution in the aflatoxin gene cluster of *Aspergillus parasiticus*. Molec. Ecol..

[38] Carbone I., Ramirez-Prado J.H., Jakobek J.L., Horn B.W. (2007). Gene duplication, modularity and adaptation in the evolution of the aflatoxin gene cluster. BMC Evol. Biol..

[39] Shaw G.J., Chick M., Hodges R. (1978). A ^13^C NMR study of the biosynthesis of the anthraquinone dothistromin by *Dothistroma pini*. Phytochemistry.

[40] Guo Y. (2008). Identification and characterization of dothistromin biosynthetic genes in the peanut pathogen *Passalora arachidicola*.

[41] Kodama M., Rose M.S., Yang G., Yun S.H., Yoder O.C., Turgeon B.G. (1999). The translocation-associated Tox1 locus of *Cochliobolus heterostrophus* is two genetic elements on two different chromosomes. Genetics.

[42] Kimura M., Tokai T., Takahashi-Ando N., Ohsato S., Fujimura M. (2007). Molecular and genetic studies of *Fusarium* trichothecene biosynthesis: Pathways, genes, and evolution. Biosci. Biotech. Biochem..

[43] Nicholson M.J., Koulman A., Monahan B.J., Pritchard B.L., Payne G.A., Scott B. (2009). Identification of two aflatrem biosynthesis gene loci in *Aspergillus flavus* and metabolic engineering of *Penicillium paxilli* to elucidate their function. Appl. Environ. Microbiol..

[44] Cary J., Ehrlich K., Beltz S., Harris-Coward P., Klich M. (2009). Characterization of the *Aspergillus ochraceoroseus* aflatoxin/sterigmatocystin biosynthetic gene cluster. Mycologia.

[45] Nierman W.C., Pain A., Anderson M.J., Wortman J.R., Kim H.S., Arroyo J., Berriman M., Abe K., Archer D.B., Bermejo C., Bennett J., Bowyer P., Chen D., Collins M., Coulsen R., Davies R., Dyer P.S., Farman M., Fedorova N., Feldblyum T.V., Fischer R., Fosker N., Fraser A., Garcia J.L., Garcia M.J., Goble A., Goldman G.H., Gomi K., Griffith-Jones S., Gwilliam R., Haas B., Haas H., Harris D., Horiuchi H., Huang J., Humphray S., Jimenez J., Keller N., Khouri H., Kitamoto K., Kobayashi T., Konzack S., Kulkarni R., Kumagai T., Lafton A., Latge J.P., Li W.X., Lord A., Majoros W.H., May G.S., Miller B.L., Mohamoud Y., Molina M., Monod M., Mouyna I., Mulligan S., Murphy L., O'Neil S., Paulsen I., Penalva M.A., Pertea M., Price C., Pritchard B.L., Quail M.A., Rabbinowitsch E., Rawlins N., Rajandream M.A., Reichard U., Renauld H., Robson G.D., de Cordoba S.R., Rodriguez-Pena J.M., Ronning C.M., Rutter S., Salzberg S.L., Sanchez M., Sanchez-Ferrero J.C., Saunders D., Seeger K., Squares R., Squares S., Takeuchi M., Tekaia F., Turner G., de Aldana C.R.V., Weidman J., White O., Woodward J., Yu J.H., Fraser C., Galagan J.E., Asai K., Machida M., Hall N., Barrell B., Denning D.W. (2005). Genomic sequence of the pathogenic and allergenic filamentous fungus *Aspergillus fumigatus*. Nature.

[46] Graziani S., Vasnier C., Daboussi M. (2004). Novel polyketide synthase from *Nectria haematococca*. Appl. Environ. Microbiol..

[47] Sakuno E., Kameyama M., Nakajima H., Yabe K. (2008). Purification and gene cloning of a dehydrogenase from *Lactobacillus brevis* that catalyzes a reaction involved in aflatoxin biosynthesis. Biosci. Biotech. Biochem..

[48] Chiang Y.-M., Szewczyk E., Davidson A.D., Entwistle R., Keller N.P., Wang C.C.C., Oakley B.R. (2010). Characterization of the *Aspergillus nidulans* monodictyphenone gene cluster. Appl. Environ. Microbiol..

[49] Kroken S., Glass N.L., Taylor J.W., Yoder O.C., Turgeon B.G. (2003). Phylogenomic analysis of type 1 polyketide synthase genes in pathogenic and saprobic ascomycetes. Proc. Natl. Acad. Sci. USA.

[50] Ehrlich K.C., Montalbano B., Boue S.M., Bhatnagar D. (2005). An aflatoxin biosynthesis cluster gene encodes a novel oxidase required for conversion of versicolorin A to sterigmatocystin. Appl. Environ. Microbiol..

[51] Henry K.M., Townsend C.A. (2005). Ordering the reductive and cytochrome P450 oxidative steps in demethylsterigmatocystin formation yields general insights into the biosynthesis of aflatoxin and related fungal metabolites. J. Am. Chem. Soc..

[52] Danks A.V., Hodges R. (1974). Polyhydroxyanthraquinones from *Dothistroma pini*. Australian J. Chem..

[53] Schwelm A. (2007). Investigation of dothistromin gene expression in *Dothistroma septosporum* and the putative role of dothistromin toxin.

[54] Bradshaw R.E., Feng Z., Schwelm A., Yang Y., Zhang S. (2009). Functional analysis of a putative dothistromin toxin MFS transporter gene. Toxins.

[55] Martin J.F., Casqueiro J., Liras P. (2005). Secretion systems for secondary metabolites: How producer cells send out messages of intercellular communication. Curr. Opin. Microbiol..

[56] Chang P.-K., Yu J., Yu J.-H. (2004). *aflT*, a MFS transporter-encoding gene located in the aflatoxin gene cluster, does not have a significant role in aflatoxin secretion. Fungal Genet. Biol..

[57] Chanda A., Roze L.V., Kang S., Artymovich K.A., Hicks G.R., Raikhel N.V., Calvo A.M., Linz J.E. (2009). A key role for vesicles in fungal secondary metabolism. Proc. Natl. Acad. Sci. USA.

[58] Ehrlich K.C., Li P., Scharfenstein L., Chang P.-K. (2010). HypC is the anthrone oxidase involved in aflatoxin biosynthesis. Appl. Environ. Microbiol..

[59] Feng Z. (2007). Further studies of dothistromin toxin genes in the fungal forest pathogen *Dothistroma septosporum*.

[60] Cary J.W., Wright M., Bhatnagar D., Lee R., Chu F.S. (1996). Molecular characterization of an *Aspergillus parasiticus* dehydrogenase gene, *norA*, located on the aflatoxin biosynthesis gene cluster. Appl. Environ. Microbiol..

[61] Ehrlich K.C., Scharfenstein L.L., Montalbano B.G., Chang P.-K. (2008). Are the genes *nadA* and *norB* involved in formation of aflatoxin G1?. Int.J. Molec. Sci..

[62] Feng G.H., Leonard T.J. (1998). Culture conditions control expression of the genes for aflatoxin and sterigmatocystin biosynthesis in *Aspergillus parasiticus* and *A. nidulans*. Appl. Environ. Microbiol..

[63] Cary J.W., Calvo A.M. (2008). Regulation of *Aspergillus* mycotoxin biosynthesis. Toxin Rev..

[64] Yu J.-H., Keller N. (2005). Regulation of secondary metabolism in filamentous fungi. Ann. Rev. Phytopathol..

[65] Zhang Y., Keller N., Tsitsigiannis D., Wilkinson H.H., An Z. (2005). Secondary metabolite gene clusters. Handbook of Industrial Mycology.

[66] Bayram O., Krappmann S., Ni M., Bok J.W., Helmstaedt K., Valerius O., Braus-Stromeyer S., Kwon N.-J., Keller N.P., Yu J.-H., Braus G.H. (2008). VelB/VeA/LaeA complex coordinates light signal with fungal development and secondary metabolism. Science.

[67] Schwelm A., Barron N.J., Zhang S., Bradshaw R.E. (2008). Early expression of aflatoxin-like dothistromin genes in the forest pathogen *Dothistroma septosporum*. Mycol. Res..

[68] Calvo A.M., Wilson R.A., Bok J.W., Keller N.P. (2002). Relationship between secondary metabolism and fungal development. Microbiol. Molec. Biol. Rev..

[69] Wilkinson H.H., Ramaswamy A., Sim S.C., Keller N.P. (2004). Increased conidiation associated with progression along the sterigmatocystin biosynthetic pathway. Mycologia.

[70] Donzelli B.G.G., Krasnoff S.B., Churchill A.C.L., Vandenberg J.D., Gibson D.M. (2010). Identification of a hybrid PKS–NRPS required for the biosynthesis of NG-391 in *Metarhizium robertsii*. Curr. Genet..

[71] Amaike S., Keller N.P. (2009). Distinct roles for VeA and LaeA in development and pathogenesis of *Aspergillus flavus*. Euk. Cell.

[72] Horowitz Brown S., Scott J.B., Bhaheetharan J., Sharpee W.C., Milde L., Wilson R.A., Keller N.P. (2009). Oxygenase coordination is required for morphological transition and the host-fungus interaction of *Aspergillus flavus*. Mol. Plant Microbe Interact..

[73] Reverberi M., Ricelli A., Zjalic S., Fabbri A.A., Fanelli C. (2010). Natural functions of mycotoxins and control of their biosynthesis in fungi. Appl. Microbiol. Biotechnol..

[74] Youngman R.J., Elstner E.F. (1984). Photodynamic and reductive mechanism of oxygen activation by the fungal phytotoxins, cercosporin and dothistromin. Oxygen Radicals in Chemistry and Biology.

[75] Ganley R.J., Newcombe G. (2006). Fungal endophytes in seeds and needles of *Pinus monticola*. Mycol. Res..

[76] Zamora P., Martinez-Ruiz C., Diez J.J. (2008). Fungi in needles and twigs of pine plantations in northern Spain. Fungal Diversity.

[77] Deckert R.J., Melville L.H., Peterson R.L. (2001). Structural features of a *Lophodermium* endophyte during the cryptic life-cycle phase in the foliage of *Pinus strobus*. Mycol. Res..

[78] Keon J., Antoniw J., Carzaniga R., Deller S., Ward J.L., Baker J.M., Beale M.H., Hammond-Kosack K., Rudd J.J. (2007). Transcriptional adaptation of *Mycosphaerella graminicola* to programmed cell death (PCD) of its susceptible wheat host. Mol. Plant Microbe Interact..

